# The Lasting Impact of Redlining on Boston Neighborhoods: Findings from the 2025 Healthy Aging Data Reports

**DOI:** 10.1093/geroni/igaf122.2591

**Published:** 2025-12-31

**Authors:** Taylor Jansen, Elizabeth Dugan

**Affiliations:** University of Massachusetts Boston, Boston, Massachusetts, United States; University of Massachusetts Boston, Boston, Massachusetts, United States

## Abstract

In 1933, the federally funded Homes Owner’s Loan Corporation (HOLC) tasked local officials to create neighborhood maps which captured credit default risk for residents applying for mortgages. Maps were color coded and assigned grades to city neighborhoods (A “Best”, B “Still Desirable”, C “Definitely Declining”, D “Hazardous”); “A” neighborhoods were often wealthy, majority White neighborhoods, while “D” neighborhoods were home to Black, immigrant residents in lower socioeconomic classes. Though outlawed in 1968, this practice has had a lasting impact on the racially segregated neighborhoods, access to fair housing, care, and health. This study used the HOLC map shapefiles provided by the University of Richmond Digital Scholarship Lab and overlayed them with current Boston neighborhood boundaries to examine if health disparities persisted in historically redlined neighborhoods. Descriptive statistics were estimated using data from the American Community Survey (ACS) (2018-2022) and the Medicare Beneficiary Summary File (MBSF) (2020-2021) by HOLC grade for 23 Boston neighborhoods. Only one neighborhood in Boston (Beacon Hill) was rated “B”, while 11 neighborhoods were rated C and D each. Comparing the “C” to “D” neighborhoods, “D” neighborhoods reported lower 65+ educational attainment, home and vehicle ownership, and median income. Historically “Hazardous” neighborhoods reported higher 65+ prevalence rates of current asthma, atrial fibrillation, chronic obstructive pulmonary disease (COPD), heart attack, liver disease, lung cancer, substance and tobacco use disorders, anxiety, and post traumatic stress disorder (PTSD). These findings underscore the detrimental and lasting effects of redlining on the health of older Boston residents, almost 100 years later.

